# Supplementary material for: Semi-Targeted Profiling of the Lipidome Changes Induced by Erysiphe Necator in Disease-Resistant and Vitis vinifera L. Varieties
Source: Int J Mol Sci. 2023 Feb 17;24(4):4072. doi: 10.3390/ijms24044072 (PMC9958630; doi:10.3390/ijms24044072)
Supplement: Supplementary file 1 [file ijms-24-04072-s001.zip › Supplementary Figure S1.pptx]

## Slide 1
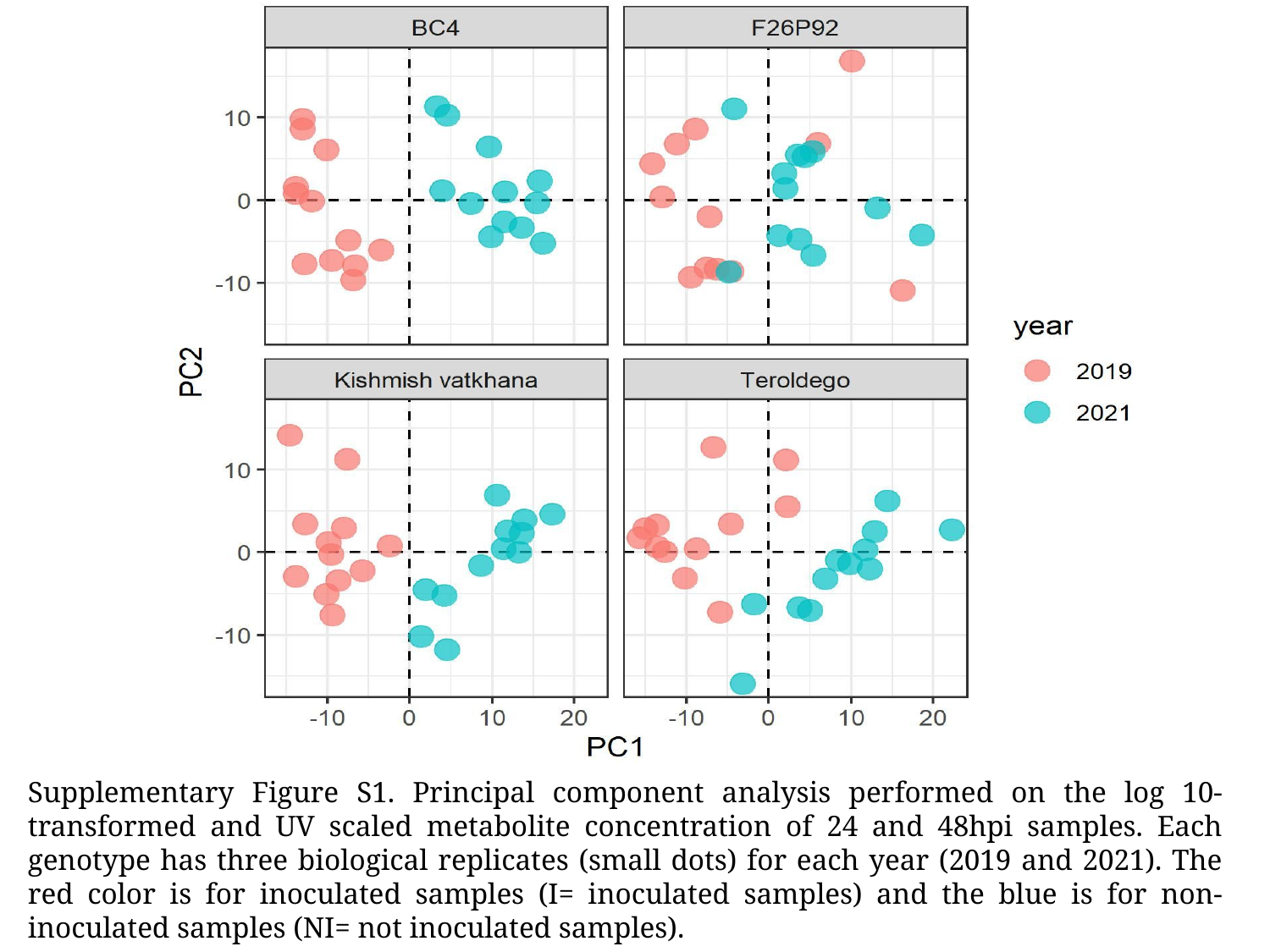

Supplementary Figure S1. Principal component analysis performed on the log 10-transformed and UV scaled metabolite concentration of 24 and 48hpi samples. Each genotype has three biological replicates (small dots) for each year (2019 and 2021). The red color is for inoculated samples (I= inoculated samples) and the blue is for non-inoculated samples (NI= not inoculated samples).
